# Discovery of Structurally Distinct Covalent KRAS G12C Inhibitor Scaffolds Through Large-Scale In Silico Screening and Experimental Validation

**DOI:** 10.3390/cancers18091367

**Published:** 2026-04-25

**Authors:** Glen J. Weiss, Joseph C. Loftus, David W. Mallery, Nhan L. Tran

**Affiliations:** 1 International Genomics Consortium, Phoenix, AZ 85014, USA; dmallery@intgen.org; 2 Department of Medicine, Chan Medical School, University of Massachusetts, Worcester, MA 01655, USA; 3 Department of Cancer Biology, Mayo Clinic Arizona, Phoenix, AZ 85054, USA; loftus.joseph@mayo.edu (J.C.L.); tran.nhan@mayo.edu (N.L.T.); 4 Department of Neurological Surgery, Mayo Clinic Arizona, Phoenix, AZ 85054, USA

**Keywords:** KRAS G12C, covalent inhibitors, switch-II pocket, virtual screening, oncology drug discovery, resistance, molecular dynamics, MM/GBSA

## Abstract

**Background/Objectives:** KRAS G12C mutations define a clinically actionable subset of solid tumors, particularly non–small cell lung cancer. Clinical responses to approved covalent inhibitors remain limited by intrinsic and acquired resistance, highlighting the need for structurally distinct inhibitor scaffolds to expand therapeutic options. The objective of this study was to identify novel covalent binders targeting the KRAS G12C switch-II pocket through large-scale in silico screening and experimental validation. **Methods:** More than 1.9 million small molecules from diverse commercial libraries were screened using covalent docking, followed by multi-stage refinement incorporating molecular dynamics simulations, MM/GBSA free-energy estimation, and cancer-focused QSAR modeling. **Results:** This integrated workflow yielded 50 prioritized compounds spanning several chemically distinct scaffold classes. These candidates displayed favorable predicted binding energetics, stable ligand-protein interactions over extended simulation timescales, and low structural similarity to clinically approved KRAS G12C inhibitors sotorasib and adagrasib. Benchmarking against these clinical agents, using identical computational parameters, yielded comparable predicted binding energies for several candidate molecules. In cellular NanoBRET target-engagement assays, selected scaffolds, including K788-7251 and AN-989/14669131, exhibited sub-micromolar engagement of KRAS G12C with minimal endothelial cytotoxicity. **Conclusions:** Collectively, these findings identify structurally distinct, KRAS G12C inhibitor chemotypes and provide tractable starting points for the development of next-generation targeted therapies.

## Simple Summary

KRAS G12C mutations occur in a substantial subset of lung, colorectal, and pancreatic cancers. Although two covalent KRAS G12C inhibitors are clinically approved, most patients develop resistance, underscoring the need for structurally diverse inhibitor scaffolds. We performed a large-scale computational screen of more than 1.9 million compounds from four commercial libraries using covalent docking (Schrödinger/Glide), molecular dynamics simulations, and MM/GBSA binding free-energy estimation. We validated selected molecules in cellular NanoBRET target-engagement and HUVEC cytotoxicity assays. Two compounds, K788-7251 and AN-989/14669131, showed sub-micromolar KRAS G12C engagement with minimal endothelial cytotoxicity, establishing them as starting points for next-generation KRAS-targeted medicinal chemistry. None of the prioritized compounds exceeded a Tanimoto similarity coefficient of 0.5 relative to sotorasib or adagrasib, confirming structural novelty.

## 1. Introduction

KRAS is among the most frequently mutated oncogenes in human cancer, driving tumorigenesis across lung, colorectal, and pancreatic malignancies through constitutive activation of downstream signaling pathways, including RAF–MEK–ERK and PI3K–AKT [1,2,3]. For decades, KRAS was widely regarded as an “undruggable” target due to its high affinity for GTP/GDP, lack of deep binding pockets, and the risk of inhibiting wild-type KRAS signaling in normal tissues [4,5,6]. This paradigm shifted with the discovery of the KRAS G12C switch-II pocket and the development of covalent inhibitors that selectively engage the mutant cysteine residue [7,8].

The clinical approval of sotorasib and adagrasib validated KRAS G12C as a druggable oncogenic driver and represented a landmark in targeted cancer therapy [9,10,11,12]. Nevertheless, clinical responses to these agents are heterogeneous and frequently transient. Both intrinsic and acquired resistance mechanisms have been documented, including secondary KRAS mutations, adaptive pathway reactivation, and tumor lineage–specific factors [13,14,15,16,17,18]. These limitations underscore the need for additional KRAS G12C inhibitor chemotypes that may differ in binding mode, kinetic properties, or resistance susceptibility.

From an oncology perspective, expanding the chemical diversity of KRAS G12C inhibitors is strategically important. Distinct scaffolds may enable alternative combination strategies, overcome specific resistance mutations, or offer differentiated pharmacokinetic or tissue distribution profiles [19,20,21,22]. However, experimental screening of millions of compounds is impractical, and rational prioritization strategies are required to efficiently identify viable candidates for preclinical testing.

In this study, we describe a large-scale, oncology-oriented discovery effort integrating covalent docking, molecular dynamics simulations, binding free-energy estimation, and cancer-specific QSAR modeling to identify novel KRAS G12C inhibitor scaffolds. Importantly, the goal of this work is not to introduce new computational chemistry methodology, but rather to apply established in silico tools in a rigorous and consistent framework to support cancer-focused compound prioritization. By benchmarking all candidates against approved KRAS G12C inhibitors using identical protocols, we provide a clinically relevant context for interpreting predicted binding performance.

## 2. Materials and Methods

### 2.1. Overall Study Design

This study employed an integrated discovery and validation strategy to identify and prioritize novel KRAS G12C inhibitor scaffolds for oncology-focused preclinical development. Large-scale in silico screening and molecular dynamics refinement were used to triage compounds for experimental testing. Selected candidates were subsequently evaluated using a NanoBRET KRAS G12C cellular target-engagement assay, along with parallel endothelial cell toxicity assessment using an MTT viability assay.

### 2.2. Compound Libraries and Initial Screening

Small-molecule libraries from ChemDiv (San Diego, CA, USA), OTAVA Chemicals (Vaughan, ON, Canada), Specs (Zoetermeer, The Netherlands), and Enamine (Kyiv, Ukraine) were screened against the KRAS G12C switch-II pocket using a covalent docking workflow. More than 1.9 million prepared ligand structures were evaluated following stereoisomer generation and protonation-state assignment. Following multi-stage computational refinement, a subset of compounds was selected for experimental testing based on predicted binding stability, chemical diversity, and commercial availability. These included representatives from multiple chemical series, such as K788-7251 (ChemDiv), AN-989/14669131 (Specs), 0118720109 (OTAVA Chemicals), and additional analogs. Sotorasib and adagrasib were purchased from MedChemExpress (Monmouth Junction, NJ, USA).

### 2.3. Protein Structure Preparation

The KRAS G12C protein structure was obtained from the Protein Data Bank (PDB ID: 6OIM), representing the GDP-bound inactive conformation with an accessible switch-II pocket. Missing residues and side chains were reconstructed, hydrogen atoms were added, and protonation states were assigned to reflect physiological pH conditions. Structural preparation was performed to ensure consistency across all docking and simulation stages, including geometry optimization and removal of crystallographic artifacts.

### 2.4. Covalent Docking

Covalent docking was conducted using a Michael addition reaction model targeting the mutant cysteine residue (Cys12). All docking calculations were performed using the Schrödinger Suite v2023-3 (Schrödinger LLC, New York, NY, USA), specifically the Glide module with a covalent docking protocol [23]. Compound libraries were prepared using LigPrep for stereoisomer enumeration and protonation-state assignment at pH 7.0 ± 1.0. The protein grid was generated centered on the Cys12 binding site within the switch-II pocket. An initial high-throughput virtual screening (HTVS) stage was applied to all prepared ligands to identify potential covalent binders, followed by standard precision (SP) docking with thorough pose refinement for top-ranked compounds. GlideScore (kcal/mol) was used as the primary ranking metric at this stage, but was not considered sufficient alone for final compound prioritization.

### 2.5. Molecular Dynamics Simulations

Top-ranked compounds were subjected to molecular dynamics (MD) simulations to assess the stability of ligand–KRAS G12C complexes over time. Short-duration simulations (1 ns) were first performed as a rapid filter to remove compounds that rapidly dissociated from the binding pocket or exhibited unstable interactions. The top 100 compounds per library were advanced to this short-simulation stage based on docking scores; from these, the top 25 per library were selected based on average MM/GBSA scores for extended simulations. Compounds that maintained stable binding during the 1 ns phase were subsequently evaluated using long (100 ns) MD simulations under physiologically relevant conditions. Simulations were performed using an explicit solvent model (TIP3P water, cubic box with minimum 10 Å buffer), physiological ionic strength (0.15 M NaCl), at 310 K (V-rescale thermostat) and 1 bar (Parrinello-Rahman barostat), with a 2-fs integration timestep. Ligand-protein complex stability was evaluated by analyzing backbone root-mean-square deviation (RMSD) and interaction stability profiles over the full simulation trajectory.

### 2.6. Binding Free-Energy Estimation

Binding free energies were estimated from MD trajectories using the molecular mechanics generalized Born surface area (MM/GBSA) method, implemented within the Schrödinger Suite. Average MM/GBSA values (kcal/mol) were computed independently from 1 ns and 100 ns simulation trajectories to assess both preliminary binding affinity and energetic convergence across simulation timescales. MM/GBSA values from the 100 ns trajectories were used as the primary ranking metric for final compound prioritization, as they better reflect equilibrated binding configurations. These values provided a more nuanced assessment of predicted binding free energy than GlideScore alone. Complete MM/GBSA data for all 50 prioritized compounds, along with both reference inhibitors, are provided in Supplementary Table S2.

### 2.7. Cancer-Focused QSAR Modeling

A cancer-focused QSAR model implemented in the Schrödinger Suite (Canvas module v5.4) was used as a supplementary ranking metric to prioritize compounds based on predicted oncology drug-likeness. This model incorporated topological, physicochemical, and fingerprint-based structural descriptors commonly associated with oncologically active small molecules. QSAR scores are reported on a normalized scale (0–1); higher scores indicate greater predicted similarity to known oncology drug candidates. The QSAR analysis was not intended to predict clinical efficacy or absolute potency, but rather to provide a complementary ranking dimension alongside structure-based metrics. QSAR scores for all 50 prioritized compounds are provided in Supplementary Table S2.

### 2.8. Benchmarking Against Approved KRAS G12C Inhibitors

Sotorasib and adagrasib were processed through the identical covalent docking, MD, and MM/GBSA workflow used for screened compounds. This ensured that all comparative assessments were methodologically consistent and provided a clinically relevant reference framework for interpreting predicted binding performance. All in silico modeling, docking, and molecular dynamics, binding free-energy estimation, and cancer-focused QSAR modeling analyses were performed by PROGEN Biyoteknoloji Ltd. Şti. (Istanbul, Turkey) under the scientific direction of Dr. Serdar Durdagi.

### 2.9. KRAS G12C NanoBRET Target Engagement Assay

Cellular KRAS G12C engagement was assessed using the NanoBRET™ Target Engagement Assay (Promega, Madison, WI, USA; catalog #CS321839). HEK293 cells were used as the host cell line and transfected using FuGENE^®^ transfection reagent (Promega, Madison, WI, USA) with the following DNA components: 8.0 μg/mL transfection carrier DNA, 1.0 μg/mL LgBiT^®^ fusion DNA, and 1.0 μg/mL SmBiT^®^ fusion DNA in Opti-MEM™ medium without phenol red (Gibco/Thermo Fisher Scientific, Waltham, MA, USA). Twenty-four hours post-transfection, test compounds were added to 96-well plates containing cells and 1× NanoBRET tracer. Plates were mixed for 15 s at 900 rpm and incubated at 37 °C with 5% CO_2_ for 1 h and 45 min. Immediately prior to luminescence measurements, 3× Complete NanoBRET Nano-Glo^®^ Substrate in Opti-MEM (50 μL per well) was added. Donor (450 nm) and acceptor (610 nm) emissions were recorded using a FLUOstar microplate reader (BMG LABTECH, Ortenberg, Germany). BRET ratios were calculated as (acceptor emission/donor emission) × 1000 to yield milliBRET units (mBU). Compound concentrations ranged from 100 pM to 10 μM. Dose–response curves were generated, and IC_50_ values were determined by nonlinear regression. Two independent plates were used for target engagement screening, with three technical replicates per concentration per plate. IC_50_ values were derived from dose–response curves by nonlinear regression.

### 2.10. Endothelial Cell Toxicity (MTT Assay)

To assess off-target cytotoxicity, human umbilical vein endothelial cells (HUVECs) were treated with the same compound concentration ranges used in the NanoBRET assay. Cell viability was measured using an MTT assay with absorbance read at 570 nm using a microplate reader. Two independent plates were analyzed for toxicity assessment, with three technical replicates per concentration per plate.

### 2.11. Cell-Based Proliferation Assays in Ba/F3 KRAS G12C Models

Cellular proliferation was evaluated using Ba/F3 murine pro–B cells engineered to express KRAS G12C and selected resistance-associated variants. Ba/F3 cells provide a well-established system for assessing oncogene dependency and inhibitor sensitivity, as their survival and proliferation are dependent on signaling from the introduced oncogenic driver [24].

Ba/F3 models included parental KRAS G12C and resistance-associated variants KRAS G12C-Q99L and KRAS G12C-R68M generated and maintained by Kyinno Biotechnology Co., Ltd. (Beijing, China). Cells were treated with compounds for 72 h across a concentration range of 10 µM to 10 nM, and viability was measured using the CellTiter-Glo^®^ luminescent assay.

To evaluate whether cellular KRAS G12C target engagement translated into functional growth inhibition, selected compounds were assessed using a 72-h CellTiter-Glo^®^ (CTG) viability assay in Ba/F3 KRAS G12C proliferation models, including parental G12C and selected resistance-associated variants. Compounds were tested across a 10 µM to 10 nM concentration range.

### 2.12. Data Analysis

IC_50_ values were calculated using dose–response curve fitting. Compounds were considered biologically active if they demonstrated sub-micromolar KRAS G12C engagement with minimal endothelial toxicity. Computational and experimental data were integrated to prioritize compounds for further preclinical development. All NanoBRET and MTT assays were performed by PROGEN Biyoteknoloji Ltd. Şti. (Istanbul, Turkey) under the scientific direction of Dr. Timuçin Avşar. CTG assays were performed by Kyinno Biotechnology Co., Ltd. (Beijing, China).

### 2.13. AI Assistance Disclosure

ChatGPT version 5.2 (OpenAI) was used to assist with manuscript organization, language refinement, formatting, and figure generation. All authors had full access to the primary experimental data and are solely responsible for data interpretation, scientific conclusions, and the final content of the manuscript.

## 3. Results

### 3.1. Identification of Structurally Distinct KRAS G12C Binder Candidates Through Large-Scale Virtual Screening

To identify novel chemotypes capable of targeting KRAS G12C, we performed a large-scale covalent virtual screening campaign focused on the switch-II pocket, the established site for clinically approved KRAS G12C inhibitors [25]. A total of 1.9 million chemically diverse small molecules from ChemDiv, Otava, Specs, and Enamine libraries were computationally prepared and screened using covalent docking. Compounds were prioritized based on their predicted ability to form a covalent Michael addition with Cys12 while adopting a sterically favorable orientation within the switch-II pocket.

**Figure 1:**
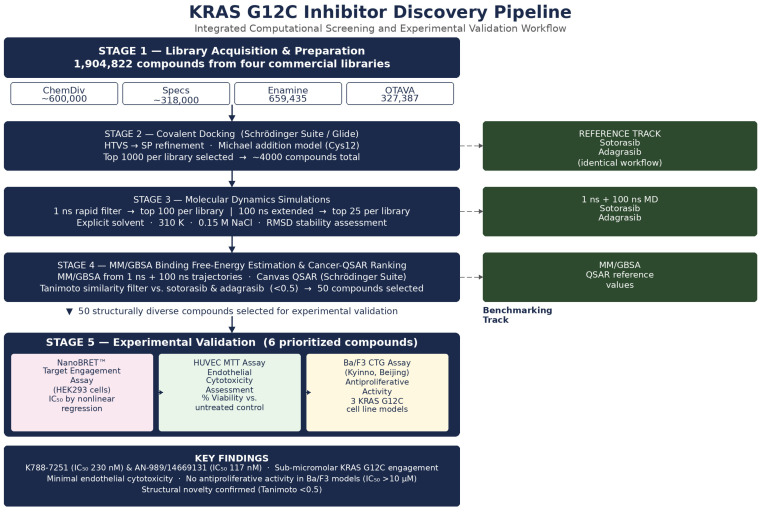
Pipeline overview flowchart. Solid arrows indicate direct sequential workflow steps; dashed arrows indicate the parallel reference/benchmarking track.

Top-ranking molecules underwent additional refinement and filtering based on docking score distributions and geometric constraints associated with the covalent adduct. Compounds exhibiting unstable or non-productive binding configurations were excluded from further analysis. This multi-stage triage process reduced the initial screening space to approximately 1000 candidates per library for subsequent molecular dynamics evaluation. The overall five-stage discovery workflow is illustrated in Figure 1. Covalent docking scores for all screened library compounds are summarized in Supplementary Table S3.

To assess the structural novelty of the prioritized compounds, chemical similarity analysis was performed relative to the clinically approved KRAS G12C inhibitors sotorasib and adagrasib. None of the selected candidates exceeded a Tanimoto similarity coefficient of 0.5, indicating that the identified molecules represent chemically distinct scaffolds rather than analogs of existing inhibitors. These results demonstrate that the screening workflow successfully enriched for structurally diverse candidate binders targeting the KRAS G12C switch-II pocket (Supplementary Figure S1).

### 3.2. Molecular Dynamics Simulations Demonstrate Stable Ligand Engagement Within the KRAS G12C Switch-II Pocket

To evaluate whether the docked complexes represented stable binding configurations rather than static docking artifacts, top-ranked compounds were subjected to molecular dynamics (MD) simulations. Initial short-duration simulations (1 ns) were used as a rapid filter to remove compounds that rapidly dissociated from the binding pocket or exhibited unstable interactions with the KRAS G12C protein.

**Figure 2:**
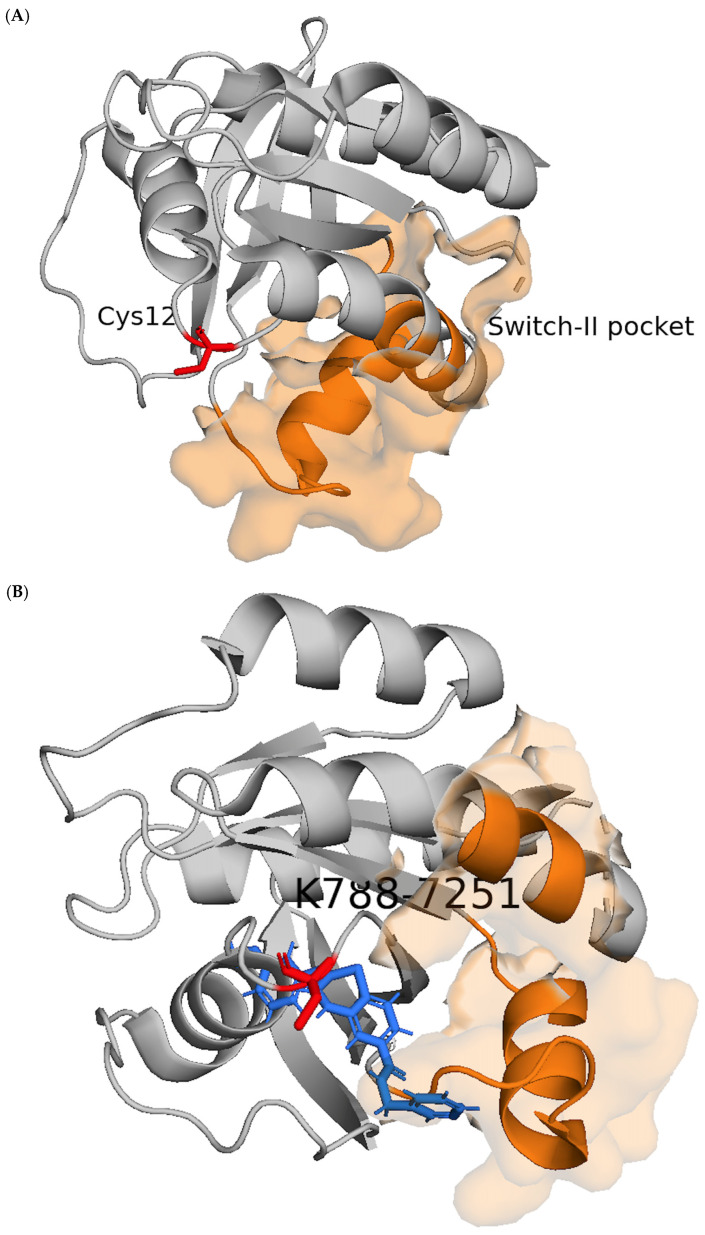

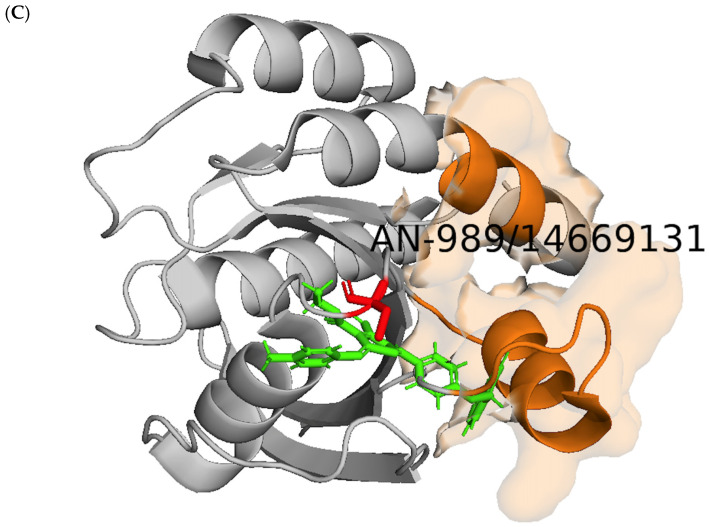
Structural context of KRAS G12C and representative inhibitor binding poses. (**A**) Structural rendering of KRAS highlighting the G12C mutation site (Cys12) and the switch-II pocket, which is the binding region targeted by covalent KRAS G12C inhibitors. The protein backbone is shown in cartoon representation, with Cys12 displayed as sticks and the switch-II pocket indicated by a semi-transparent surface. (**B**,**C**) Representative binding poses of K788-7251 (**B**) and AN-989/14669131 (**C**) within the KRAS G12C switch-II pocket. The ligands are shown in stick representation, positioned within the pocket adjacent to Cys12 to illustrate potential pocket occupancy and scaffold placement. Ligand poses are shown for illustrative purposes to highlight pocket occupancy and structural diversity of the candidate inhibitors rather than to represent precise binding geometries. Protein backbone shown in grey; switch-II pocket in orange semi-transparent surface; Cys12 highlighted in red; K788-7251 ligand in blue; AN-989/14669131 ligand in green.

Compounds that maintained stable binding during the short simulation phase were subsequently evaluated using extended 100 ns MD simulations under physiologically relevant conditions. Several candidates exhibited persistent interactions with key switch-II pocket residues, maintaining covalent attachment to Cys12 while preserving stabilizing interactions within the surrounding residues. Analysis of root-mean-square deviation (RMSD) and interaction stability profiles confirmed that the selected ligands maintained consistent conformational positioning within the pocket throughout the simulation period (Supplementary Figure S2). Representative structural renderings illustrating the KRAS G12C switch-II pocket, and representative inhibitor poses are shown in Figure 2A–C. These findings support the structural plausibility of the predicted binding modes and suggest that the identified compounds form stable KRAS G12C complexes.

### 3.3. Binding Free-Energy Analysis Supports Favorable Predicted Affinity of Candidate Compounds

To further evaluate binding free energetics, molecular mechanics generalized Born surface area (MM/GBSA) calculations were performed using trajectories derived from both short (1 ns) and extended (100 ns) MD simulations. Average binding free-energy estimates were calculated to assess both predicted affinity and energetic convergence across simulation timescales.

Several compounds demonstrated favorable MM/GBSA values, comparable to or exceeding those for sotorasib and adagrasib, when processed using the same computational workflow. The use of identical simulation parameters provided an internally controlled benchmarking framework for interpreting predicted binding energies.

Compounds showing consistent MM/GBSA stability across simulation durations were prioritized for experimental validation, as this convergence suggests energetically stable interactions within the KRAS G12C switch-II pocket.

### 3.4. Cancer-Focused QSAR Modeling Refines Candidate Ranking

To complement the structure-based prioritization strategy, a cancer-focused QSAR model was applied to further rank candidate compounds. This model incorporated physicochemical descriptors and structural features commonly associated with oncology drug candidates, including parameters related to molecular complexity, drug-likeness, and pharmacologically relevant chemical motifs.

QSAR scores were not intended to predict clinical efficacy but rather to provide an additional ranking metric for selecting chemically tractable compounds for experimental testing. Compounds advancing to cellular assays generally exhibited moderate-to-high QSAR scores relative to the broader screened population, indicating that the integrated computational workflow enriched for molecules with favorable properties for oncology-focused discovery.

### 3.5. Computational Benchmarking Against Clinically Approved KRAS G12C Inhibitors

To validate the computational pipeline, sotorasib and adagrasib were processed through the identical covalent docking, MD, and MM/GBSA workflow used for screened compounds. Both reference inhibitors exhibited stable binding in the switch-II pocket and favorable predicted binding energies, consistent with their known biochemical activity.

Notably, several newly identified compounds demonstrated predicted binding energies comparable to those of these clinical agents. This benchmarking ensured that candidate prioritization was grounded in clinically relevant reference standards and supported the advancement of selected scaffolds to experimental evaluation.

### 3.6. Cellular KRAS G12C Target Engagement and Endothelial Safety Profile

Selected compounds were next evaluated for their ability to engage KRAS G12C in a cellular context using a NanoBRET target engagement assay in HEK293 cells. This assay directly measures intracellular binding to KRAS G12C and provides orthogonal validation of computational predictions. Multiple structurally distinct scaffolds demonstrated measurable target engagement, with several achieving sub-micromolar potency (Table 1). Among the most active compounds, AN-989/14669131 exhibited potency comparable to the reference inhibitor sotorasib (117 nM vs. 116 nM), while K788-7251 demonstrated consistent sub-micromolar activity (230 nM). As expected, adagrasib displayed the highest potency among the compounds tested (20 nM), serving as a low-nanomolar benchmark control. Dose–response curves for all tested compounds are provided in Supplementary Figure S3.

Additional compounds showed moderate activity, including 0118720109 (1.24 µM) and 0118720069 (2.47 µM), indicating graded target engagement across chemically diverse scaffold classes.

To assess whether KRAS G12C engagement was associated with nonspecific cytotoxicity, compounds were evaluated in parallel using an MTT viability assay in human umbilical vein endothelial cells (HUVECs). Endothelial cells were selected as a representative non-transformed cell type to assess potential off-target toxicity. Compounds demonstrating KRAS G12C engagement maintained high endothelial viability across the tested concentration range. Notably, K788-7251 and AN-989/14669131 showed minimal cytotoxicity at concentrations exceeding their respective NanoBRET IC_50_ values, indicating that their observed target engagement was unlikely to result from generalized cellular toxicity. These results support the presence of a preliminary therapeutic window for the prioritized scaffolds.

### 3.7. Integration of Computational and Experimental Prioritization Screening Identifies Lead KRAS G12C Chemotypes

The integrated computational pipeline successfully enriched for compounds demonstrating measurable cellular KRAS G12C engagement. Although the limited number of experimentally tested compounds precluded formal statistical correlation analysis, compounds exhibiting favorable docking scores, sustained molecular dynamics stability, and consistent MM/GBSA free-energy profiles were more likely to demonstrate measurable NanoBRET activity.

Among the evaluated molecules, K788-7251 and AN-989/14669131 emerged as representative scaffolds combining favorable predicted binding energetics, sub-micromolar cellular activity, and minimal endothelial toxicity. These compounds were therefore selected as illustrative examples for structural modeling and as starting points for further medicinal chemistry optimization.

### 3.8. Antiproliferative Activity in Ba/F3 KRAS G12C Proliferation Models

To determine whether KRAS G12C binding translated into functional pathway inhibition, selected compounds were evaluated in oncogene-dependent Ba/F3 proliferation models.

To evaluate whether cellular KRAS G12C target engagement translated into functional growth inhibition, selected compounds were assessed using a 72-h CellTiter-Glo^®^ (CTG) viability assay in Ba/F3 KRAS G12C proliferation models, including parental G12C and selected resistance-associated variants. These Ba/F3 models represent engineered oncogene-dependent systems rather than human tumor-derived cell lines.

Compounds were tested across a 10 µM to 10 nM concentration range. As expected, the reference inhibitor sotorasib produced marked, dose-dependent inhibition across all three Ba/F3 KRAS G12C models, with IC50 values ranging from low micromolar to submicromolar (Supplementary Table S1). In contrast, early library compounds K788-7251 and AN-989/14669131 did not demonstrate measurable antiproliferative activity within the tested concentration range (≤10 µM), and IC_50_ values could not be reliably determined. These results indicate that, despite measurable KRAS G12C engagement in NanoBRET assays, selected early library hits did not translate into functional growth suppression in oncogene-dependent proliferation models under the conditions tested.

## 4. Discussion

The present study identifies structurally distinct KRAS G12C inhibitor scaffolds through an integrated computational-experimental discovery strategy. Table 1 summarizes the computational and experimental characteristics of the leading compounds, including predicted binding metrics, NanoBRET IC_50_ values, and endothelial toxicity assessment. Several prioritized molecules, including K788-7251 and AN-989/14669131, demonstrated sub-micromolar cellular KRAS G12C engagement while maintaining minimal endothelial cytotoxicity, suggesting a favorable preliminary safety profile. Importantly, these compounds exhibit low structural similarity to clinically approved KRAS G12C inhibitors, indicating that the screening framework successfully expanded the chemical diversity of KRAS-targeting scaffolds. Together, these findings highlight the feasibility of integrating large-scale computational screening with cellular validation to rapidly identify novel candidate chemotypes for further optimization.

Covalent KRAS G12C inhibitors have transformed the therapeutic landscape for a subset of KRAS-mutant cancers, particularly non–small cell lung cancer (NSCLC) [1,2,3]. The clinical success of sotorasib and adagrasib has validated KRAS G12C as a druggable oncogenic driver; however, responses remain heterogeneous and frequently transient [4,7,9,10,26]. Both intrinsic and acquired resistance mechanisms, including secondary KRAS mutations, adaptive pathway reactivation, and bypass signaling through receptor tyrosine kinases, limit the durability of therapeutic responses. These limitations underscore the need to expand the structural and mechanistic diversity of KRAS G12C inhibitors, which may enable alternative binding interactions, distinct pharmacologic properties, and improved compatibility with rational combination strategies [11,13,14,27].

To address this challenge, we implemented a multi-stage discovery pipeline integrating large-scale covalent docking, MD simulations, MM/GBSA free-energy estimation, cancer-focused QSAR modeling, and benchmarking against approved inhibitors. Screening more than 1.9 million compounds enabled exploration of an expansive chemical space while computational triage reduced this pool to a manageable set of candidates for experimental validation. MD simulations allowed dynamic assessment of ligand stability within the KRAS switch-II pocket beyond static docking poses, while MM/GBSA calculations provided comparative estimates of binding energetics [28]. Importantly, sotorasib and adagrasib were processed through the same computational workflow, allowing predicted binding energies of candidate molecules to be interpreted relative to clinically validated reference inhibitors.

Experimental validation confirmed that several computationally prioritized molecules bind KRAS G12C in cell models. NanoBRET assays demonstrated measurable intracellular binding for multiple scaffolds, including K788-7251 and AN-989/14669131, both of which exhibited sub-micromolar target binding. These findings are significant because predicted binding affinity does not always translate into intracellular target engagement due to limitations in membrane permeability, intracellular exposure, or competition with endogenous protein–protein interaction. The observation that multiple compounds retained measurable cellular binding, therefore, provides strong validation of the computational prioritization strategy and supports the translational relevance of the discovery pipeline.

A key objective of this work was to broaden the structural landscape of KRAS G12C inhibitors. Current clinically approved agents share common structural features and rely on covalent engagement of Cys12 within the switch-II pocket [15,16,19,29]. While this strategy confers mutant selectivity, it also creates shared vulnerabilities to resistance. Preclinical studies have shown that distinct KRAS G12C inhibitor chemotypes can exhibit differential susceptibility to resistance mutations and may produce distinct downstream signaling effects. The identification of chemically independent scaffolds in this study, therefore, represents an important step toward diversifying the pharmacological compounds available for KRAS-targeted therapy [4,7,9,10,20].

Functional proliferation assays in Ba/F3 KRAS G12C models revealed a divergence between cellular target binding and antiproliferative activity for early screening hits. Although K788-7251 and AN-989/14669131 demonstrated measurable cellular KRAS G12C binding in NanoBRET assays, they did not suppress proliferation in oncogene-dependent Ba/F3 models at concentrations up to 10 µM. In contrast, sotorasib produced robust dose-dependent inhibition of proliferation. This discrepancy likely reflects differences in intracellular environment, covalent reaction kinetics, pathway suppression efficiency, or cellular dependency on KRAS signaling in the cell-based assay. These observations suggest the complexity of translating biochemical approaches into functional pathway inhibition and highlight the need for further medicinal chemistry optimization.

Safety considerations represent another important limitation in early oncology drug discovery. Endothelial cell viability assays indicated minimal cytotoxicity for compounds demonstrating KRAS G12C engagement, suggesting that the observed activity was not driven by nonspecific cellular toxicity. Although such assays cannot predict clinical tolerability, they provide an initial indication that these scaffolds may possess a preliminary therapeutic window suitable for further development.

This study has several limitations. Biological evaluation was limited to in vitro assays, and a comprehensive assessment of antitumor activity will require testing in vivo. Additionally, predicted binding energetics derived from computational modeling cannot substitute for experimentally measured biochemical potency. Finally, although several compounds demonstrated cellular KRAS G12C binding, their lack of antiproliferative activity in Ba/F3 models highlights the complexity of linking target engagement to functional pathway inhibition. We emphasize the need for further structural optimization.

Despite these limitations, this work demonstrates that an integrated computational–experimental discovery framework can efficiently identify structurally novel KRAS G12C inhibitor scaffolds capable of engaging the target protein in cells. By expanding the chemical diversity of KRAS-targeted compounds, this approach provides new starting points for the development of next-generation inhibitors designed to overcome resistance, refine pharmacologic properties, and support rational combination therapies. Continued optimization and biological evaluation of these chemotypes may ultimately contribute to improving long-term outcomes for patients with KRAS-mutant cancer [30].

## 5. Conclusions

This study demonstrates that an integrated computational–experimental discovery framework can efficiently identify structurally novel KRAS G12C inhibitor scaffolds from a large chemical space. Covalent virtual screening of more than 1.9 million compounds using the Schrödinger Suite, refined by molecular dynamics simulations, MM/GBSA free-energy estimation, and cancer-focused QSAR modeling, yielded 50 prioritized candidates spanning four chemically distinct scaffold classes. Structural novelty was confirmed by Tanimoto similarity analysis, with no candidate exceeding a coefficient of 0.5 relative to sotorasib or adagrasib. Among experimentally evaluated compounds, K788-7251 and AN-989/14669131 demonstrated sub-micromolar cellular KRAS G12C engagement with minimal endothelial cytotoxicity, supporting their advancement as starting points for medicinal chemistry optimization. Although current hits did not suppress proliferation in oncogene-dependent Ba/F3 models at concentrations up to 10 µM, this disconnect highlights important directions for structural refinement and mechanistic follow-up, including confirmation of covalent bond formation and optimization of intracellular potency. Expanding the chemical diversity of KRAS G12C inhibitors may ultimately enable next-generation therapies with improved efficacy against resistance-conferring mutations and broader applicability across KRAS-driven malignancies.

## Figures and Tables

**Table 1:** Cellular KRAS G12C target engagement and endothelial toxicity profile.

Compound ID	Vendor	IC_50_	Relative Potency vs. Sotorasib	HUVEC Toxicity (MTT)
**K788-7251**	ChemDiv	230 nM	~2.0× less potent	Minimal
**AN-989/14669131**	Specs	117 nM	Comparable	Minimal
**Sotorasib**	Medchemexpress	116 nM	Reference	Minimal
**Adagrasib**	Medchemexpress	20 nM	~6× more potent	Minimal
**0118720109**	OTAVA Chemicals	1.24 µM	~10× less potent	Minimal
**0118720069**	OTAVA Chemicals	2.47 µM	~21× less potent	Minimal

## Data Availability

All data generated or analyzed during this study are included in this published article and its Supplementary Information Files. Additional raw datasets supporting the conclusions of this study are available from the corresponding author upon reasonable request.

## Supplementary Materials

The following supporting information can be downloaded at https://www.mdpi.com/article/10.3390/cancers18091367/s1, Figure S1: NanoBRET™ KRAS G12C target engagement dose–response curves for experimentally validated compounds; Figure S2: HUVEC endothelial cell viability for tested compounds; Figure S3: Chemical structures of experimentally tested compounds; Table S1: Ba/F3 KRAS G12C antiproliferative activity; Table S2: In silico results: docking scores, MM/GBSA binding free-energy estimates, and cancer-focused QSAR scores for all 50 prioritized compounds and reference inhibitors; Table S3: NanoBRET™ target engagement assay results.

## References

[1] Skoulidis F., Li B.T., Dy G.K., Price T.J., Falchook G.S., Wolf J., Italiano A., Schuler M., Borghaei H., Barlesi F. (2021). Sotorasib for Lung Cancers with KRAS p.G12C Mutation. N. Engl. J. Med..

[2] Jänne P.A., Riely G.J., Gadgeel S.M., Heist R.S., Ou S.I., Pacheco J.M., Johnson M.L., Sabari J.K., Leventakos K., Yau E. (2022). Adagrasib in Non–Small-Cell Lung Cancer Harboring a KRAS^G12C^ Mutation. N. Engl. J. Med..

[3] Canon J., Rex K., Saiki A.Y., Mohr C., Cooke K., Bagal D., Gaida K., Holt T., Knutson C.G., Koppada N. (2019). The Clinical KRAS(G12C) Inhibitor AMG 510 Drives Anti-Tumour Immunity. Nature.

[4] Awad M.M., Liu S., Rybkin I.I., Arbour K.C., Dilly J., Zhu V.W., Johnson M.L., Heist R.S., Patil T., Riely G.J. (2021). Acquired Resistance to KRAS^G12C^ Inhibition in Cancer. N. Engl. J. Med..

[5] Boriack-Sjodin P.A., Margarit S.M., Bar-Sagi D., Kuriyan J. (1998). The Structural Basis of the Activation of Ras by Sos. Nature.

[6] Pantsar T. (2020). The Current Understanding of KRAS Protein Structure and Dynamics. Comput. Struct. Biotechnol. J..

[7] Xue J.Y., Zhao Y., Aronowitz J., Mai T.T., Vides A., Qeriqi B., Kim D., Li C., de Stanchina E., Greco L. (2020). Rapid Non-Uniform Adaptation to Conformation-Specific KRAS(G12C) Inhibition. Nature.

[8] Hunter J.C., Gurbani D., Ficarro S.B., Carrasco M.A., Lim S.M., Choi H.G., Xie T., Marto J.A., Chen Z., Gray N.S. (2014). In Situ Selectivity Profiling and Crystal Structure of SML-8-73-1, an Active Site Inhibitor of Oncogenic K-Ras G12C. Proc. Natl. Acad. Sci. USA.

[9] Tanaka N., Lin J.J., Li C., Ryan M.B., Zhang J., Kiedrowski L.A., Michel A.G., Syed M.U., Fella K.A., Sakhi M. (2021). Clinical Acquired Resistance to KRAS^G12C^ Inhibition through a Novel KRAS Switch-II Pocket Mutation and Polyclonal Alterations Converging on RAS–MAPK Reactivation. Cancer Discov..

[10] Hallin J., Engstrom L.D., Hargis L., Calinisan A., Aranda R., Briere D.M., Sudhakar N., Bowcut V., Baer B.R., Ballard J.A. (2020). The KRAS^G12C Inhibitor MRTX849 Provides Insight Toward Therapeutic Susceptibility of KRAS-Mutant Cancers in Mouse Models and Patients. Cancer Discov..

[11] Moore A.R., Rosenberg S.C., McCormick F., Malek S. (2020). RAS-Targeted Therapies: Is the Undruggable Drugged?. Nat. Rev. Drug Discov..

[12] Hong D.S., Fakih M.G., Strickler J.H., Desai J., Durm G.A., Shapiro G.I., Falchook G.S., Price T.J., Sacher A., Denlinger C.S. (2020). KRAS^G12C^ Inhibition with Sotorasib in Advanced Solid Tumors. N. Engl. J. Med..

[13] Ryan M.B., Fece de la Cruz F., Phat S., Myers D.T., Wong E., Shahzade H.A., Hong C.B., Corcoran R.B. (2020). Vertical Pathway Inhibition Overcomes Adaptive Feedback Resistance to KRAS^G12C^ Inhibition. Clin. Cancer Res..

[14] Fakih M.G., Kopetz S., Kuboki Y., Kim T.W., Munster P.N., Ramalingam S.S., Strickler J.H., Kato T., Borg C., Gollerkeri A. (2022). Sotorasib for Previously Treated Colorectal Cancers with KRAS^G12C^ Mutation (CodeBreaK100): A Multicentre, Multi-Cohort, Open-Label, Phase 2 Trial. Lancet Oncol..

[15] Ostrem J.M., Peters U., Sos M.L., Wells J.A., Shokat K.M. (2013). K-Ras(G12C) Inhibitors Allosterically Control GTP Affinity and Effector Interactions. Nature.

[16] Patricelli M.P., Janes M.R., Li L.S., Hansen R., Peters U., Kessler L.V., Chen Y., Kucharski J.M., Feng J., Zarrinkar P.P. (2016). Selective Inhibition of Oncogenic KRAS Output with Small Molecules Targeting the Inactive State. Cancer Discov..

[17] Koga T., Suda K., Fujino T., Ohara S., Hamada A., Nishino M., Chiba M., Shimoji M., Takemoto T., Arita T. (2021). KRAS Secondary Mutations That Confer Acquired Resistance to KRAS G12C Inhibitors, Sotorasib and Adagrasib, and Overcoming Strategies: Insights from In Vitro Experiments. J. Thorac. Oncol..

[18] Zhao Y., Murciano-Goroff Y.R., Xue J.Y., Ang A., Lucas J., Mai T.T., Da Cruz Paula A.F., Saiki A.Y., Mohn D., Achanta P. (2021). Diverse Alterations Associated with Resistance to KRAS(G12C) Inhibition. Nature.

[19] Lito P., Solomon M., Li L.S., Hansen R., Rosen N. (2016). Allele-Specific Inhibitors Inactivate Mutant KRAS G12C by a Trapping Mechanism. Science.

[20] Amodio V., Yaeger R., Arcella P., Cancelliere C., Lamba S., Lorenzato A., Arena S., Montone M., Mussolin B., Bian Y. (2020). EGFR Blockade Reverts Resistance to KRAS^G12C^ Inhibition in Colorectal Cancer. Cancer Discov..

[21] Ruess D.A., Heynen G.J., Ciecielski K.J., Ai J., Berninger A., Kabacaoglu D., Görgülü K., Dantes Z., Wörmann S.M., Diakopoulos K.N. (2018). Mutant KRAS-Driven Cancers Depend on PTPN11/SHP2 Phosphatase. Nat. Med..

[22] Nagasaka M., Li Y., Sukari A., Ou S.I., Al-Hallak M.N., Nagasaka A. (2020). KRAS G12C Game of Thrones, Which Direct KRAS Inhibitor Will Claim the Iron Throne?. Cancer Treat. Rev..

[23] Fell J.B., Fischer J.P., Baer B.R., Blake J.F., Bouhana K., Briere D.M., Brown K.D., Burgess L.E., Burns A.C., Burkard M.R. (2020). Identification of the Clinical Development Candidate MRTX849, a Covalent KRAS^G12C^ Inhibitor for the Treatment of Cancer. J. Med. Chem..

[24] Warmuth M., Kim S., Gu X., Xia G., Adrián F. (2007). Ba/F3 Cells and Their Use in Kinase Drug Discovery. Curr. Opin. Oncol..

[25] Janes M.R., Zhang J., Li L.S., Hansen R., Peters U., Guo X., Chen Y., Babbar A., Firdaus S.J., Darjania L. (2018). Targeting KRAS Mutant Cancers with a Covalent G12C-Specific Inhibitor. Cell.

[26] de Langen A.J., Johnson M.L., Mazieres J., Dingemans A.M.C., Mountzios G., Pless M., Wolf J., Schuler M., Lena H., Skoulidis F. (2023). Sotorasib versus Docetaxel for Previously Treated Non-Small-Cell Lung Cancer with KRAS^G12C^ Mutation: A Randomised, Open-Label, Phase 3 Trial. Lancet.

[27] Gilmartin A.G., Bleam M.R., Groy A., Moss K.G., Minthorn E.A., Kulkarni S.G., Rominger C.M., Erskine S., Fisher K.E., Yang J. (2011). GSK1120212 (JTP-74057) Is an Inhibitor of MEK Activity and Activation with Favorable Pharmacokinetic Properties for Sustained In Vivo Pathway Inhibition. Clin. Cancer Res..

[28] Kim D., Xue J.Y., Lito P. (2020). Targeting KRAS(G12C): From Inhibitory Mechanism to Modulation of Antitumor Effects in Patients. Cell.

[29] Kettle J.G., Bagal S.K., Bickerton S., Bodnarchuk M.S., Boyd S., Breed J., Carbajo R.J., Cassar D.J., Chakraborty A., Cosulich S. (2022). Discovery of AZD4625, a Covalent Allosteric Inhibitor of the Mutant GTPase KRAS^G12C^. J. Med. Chem..

[30] Saleh K., Kordahi M., Felefly T., Kourie H.R., Khalife N. (2021). KRAS-Targeted Therapies in Advanced Solid Cancers: Drug the Undruggable?. Pharmacogenomics.

