# Supplementary material for: Discovery of Structurally Distinct Covalent KRAS G12C Inhibitor Scaffolds Through Large-Scale In Silico Screening and Experimental Validation
Source: Cancers (Basel). 2026 Apr 25;18(9):1367. doi: 10.3390/cancers18091367 (PMC13162765; doi:10.3390/cancers18091367)
Supplement: Supplementary file 1 [file cancers-18-01367-s001.zip › Supplementary Figure S3.pdf]

Chemical Structures of Tested Compounds

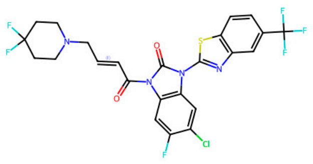

Sotorasib
MedChemExpress

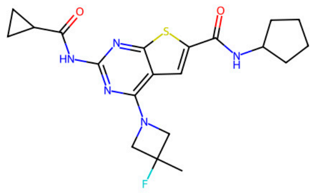

Adagrasib
MedChemExpress

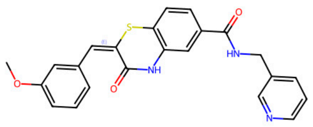

K788-7251
ChemDiv

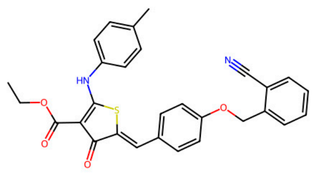

AN-989/14669131
Specs

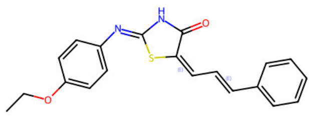

0118720109
OTAVA Chemicals

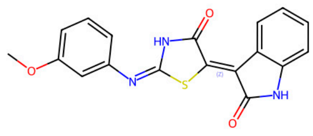

0118720069
OTAVA Chemicals
