# Supplementary figures and images for: Discovery of Structurally Distinct Covalent KRAS G12C Inhibitor Scaffolds Through Large-Scale In Silico Screening and Experimental Validation
Source: Cancers (Basel). 2026 Apr 25;18(9):1367. doi: 10.3390/cancers18091367 (PMC13162765; doi:10.3390/cancers18091367)

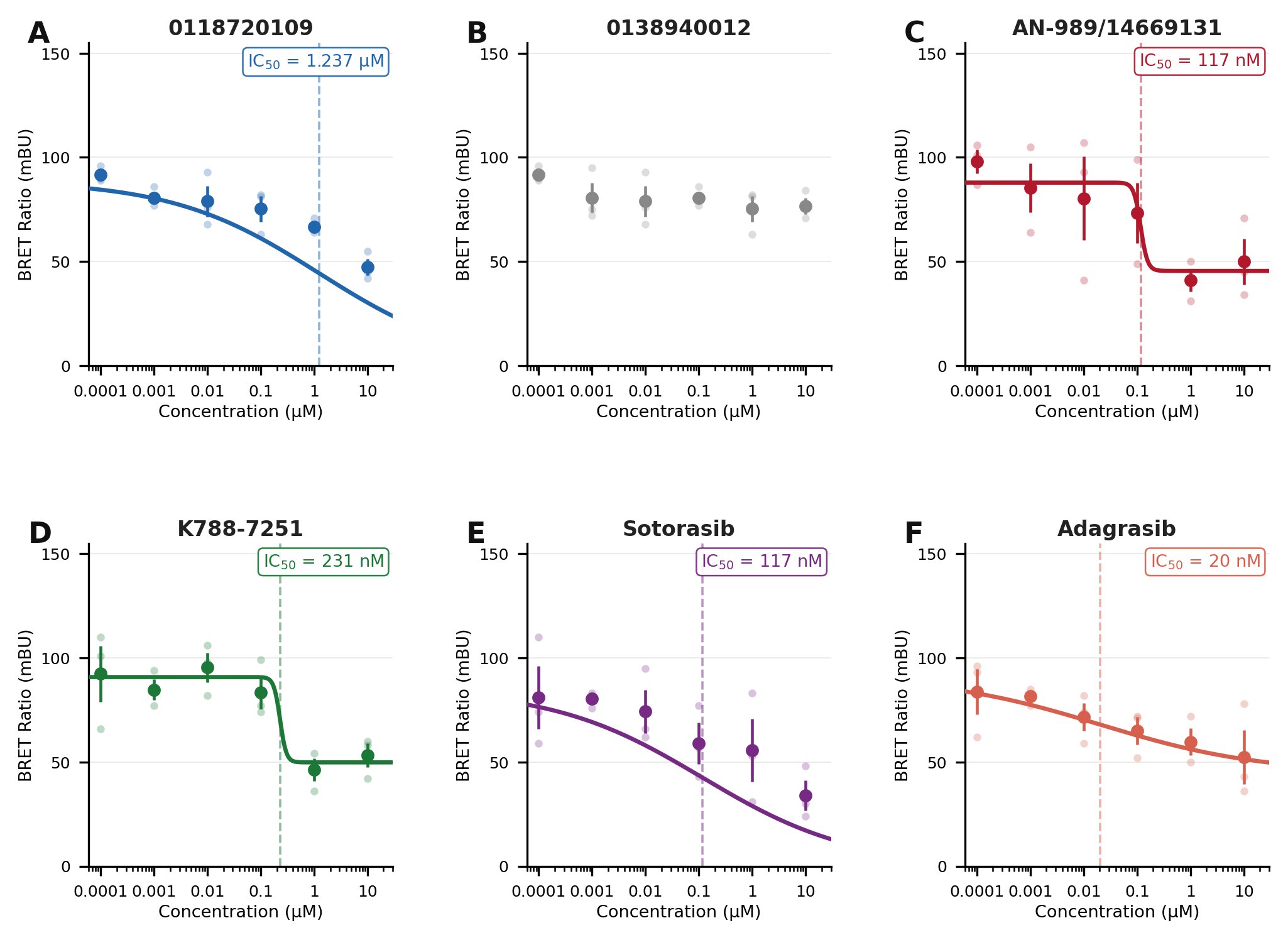

Supplement: Supplementary file 1 [file cancers-18-01367-s001.zip › Supplementary_Figure_S1.png]

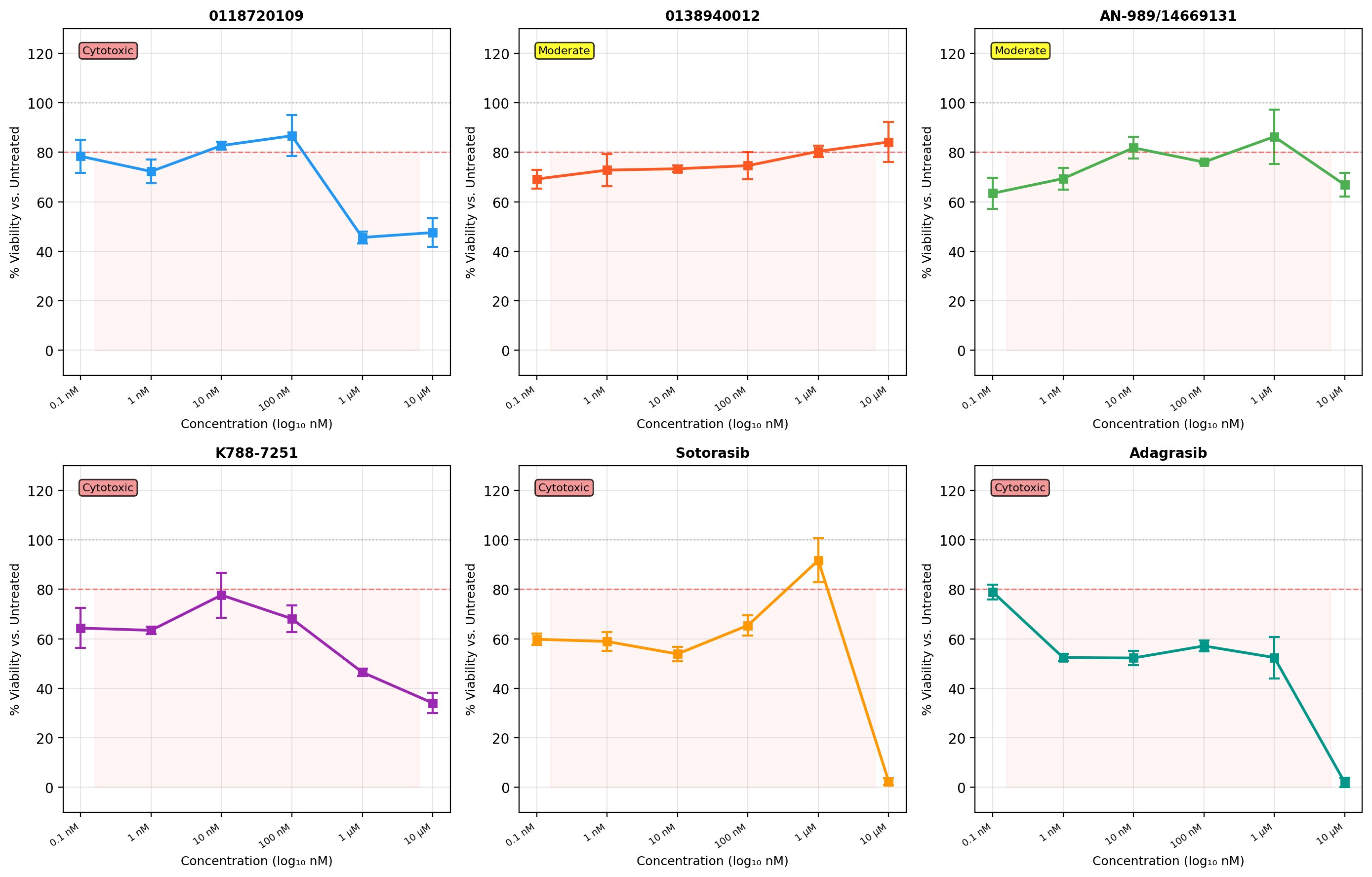

Supplement: Supplementary file 1 [file cancers-18-01367-s001.zip › Supplementary_Figure_S2.png]
